# PREVENTion and treatment of incontinence-associated dermatitis through a codesigned manual (PREVENT-IAD): a study protocol for a feasibility cluster randomised controlled trial with a nested process evaluation

**DOI:** 10.1136/bmjopen-2024-092338

**Published:** 2024-12-23

**Authors:** Sue Woodward, Tanya Graham, Sangeeta Sooriah, Dimitri Beeckman, Chris Chatterton, Mandy Fader, Francesca Fiorentino, Joanne M Fitzpatrick, Ruth Harris, Jan Kottner, Christine Norton, Peter Worsley

**Affiliations:** 1 Florence Nightingale Faculty of Nursing, Midwifery and Palliative Care, King's College London, London, UK; 2 Healthwatch Camden, London, UK; 3 Public Health and Primary Care, Ghent University, Gent, Belgium; 4 School of Health Sciences, Örebro University, Örebro, Sweden; 5 Faculty of Medicine, Nursing and Health Sciences, Monash University, Melbourne, Victoria, Australia; 6 Freelance Academic and Researcher, Patient Advocate, London, UK; 7 School of Health Sciences, University of Southampton, Southampton, Hampshire, UK; 8 LICTR, University of Leeds Leeds Institute of Clinical Trials Research, Leeds, UK; 9 Institute for Clinical Nursing Science, Charité-Universitätsmedizin Berlin, Berlin, Germany

**Keywords:** DERMATOLOGY, Clinical Trial, WOUND MANAGEMENT, Feasibility Studies, Urinary incontinences

## Abstract

**Introduction:**

Incontinence is commonly experienced by adults who receive care support in a residential facility or in their own home. These individuals are at risk of developing incontinence‐associated dermatitis (IAD), which is caused by prolonged and repeated exposure of the skin to urine or faeces. An IAD manual was developed providing an evidence-based clinical algorithm and an e-learning training programme for the prevention and treatment of IAD. The aim of the study is to establish the feasibility of conducting a definitive trial to examine the clinical and cost-effectiveness of the IAD manual. The objectives are to assess recruitment and attrition rates, acceptability of the IAD manual and intervention fidelity.

**Methods and analysis:**

A feasibility cluster randomised controlled trial will be conducted in residential nursing homes and in the homes of people receiving formal care support in London and Hampshire, England. A total of six clusters including n=248 participants who are incontinent of urine, or faeces will be included. At each intervention site, care staff will be trained to implement the IAD manual over a 6-month period. Quantitative outcomes include IAD incidence and severity, IAD-related pain, satisfaction with care and mental health. A qualitative evaluation of care staff and care receivers’ experiences of participation will be conducted. Rates and proportions of each feasibility outcome will be described informing the sample size estimation for a definitive cluster randomised controlled trial. A thematic analysis of the qualitative data will be guided by a logic model detailing potential factors impacting on both the study methodology and adoption of the IAD manual into routine care.

**Ethics and dissemination:**

The study received the approval of the Queens Square Ethics Committee Health Research Authority 23/LO/036, (Project ID 296167). Results will be disseminated through peer-reviewed open-access journals and international conferences

**Trial registration number:**

ISRCTN70866724.

**STRENGTHS AND LIMITATIONS OF THIS STUDY**

- The trial procedures and intervention have been developed from current evidence and logic modelling with sustained involvement from people with lived experience, informal carers and care staff who manage skincare for people at risk/with incontinence‐associated dermatitis (IAD).
- A mixed methods approach will be used to investigate the feasibility and acceptability of both the intervention and the planned study procedures.
- The study is embedded within the context of care home and home care agency provision enabling an understanding of how the intervention may be implemented in community and social care settings.
- The study design is a cluster randomised controlled trial to minimise potential bias and contamination.
- The study will be conducted in two regions in England which may limit the generalisability of the study findings.

## Introduction

### Background

 Incontinence‐associated dermatitis (IAD) is an irritant contact dermatitis caused by prolonged and repeated exposure of the skin to urine, faeces, or both.^1^ It is characterised by erythema, maceration and in some cases skin loss, swelling and bullae.^2^ IAD can be painful and is often associated with itch. If not prevented or treated, IAD can lead to secondary infections, increased risk of pressure ulcer development and morbidity.^3^ This can impact quality of life, such as reduced social activities and a greater dependence on formal and informal carers.

Prevalence and incidence estimates for IAD among people receiving long-term care are few and variable with no reliable UK estimates available. In a long-term care facility in the USA, IAD prevalence of 23% on admission with 8% incidence (in those without IAD at admission) was reported over 12 weeks.^4^ IAD prevalence among those with incontinence could well be higher in the community (41%)^5^ although the proportion of people experiencing incontinence is lower (~35%)^6^ than in nursing care homes (43%–77%—mean 58%).^7^ This may be due to different skincare routines or lack of support with personal hygiene at the time it is needed in the community. Among community-dwelling adults with faecal incontinence, a prevalence of IAD of 51% has been reported.^8^ Recent studies in nursing care homes in Germany estimate the point prevalence of IAD at 20.1%^9^ and IAD incidence of 23%–26% within 6 months.^10^ In the USA, the incidence of IAD was found to be 5.5% over 14 days, among nursing home residents^11^ and as high as 30% over 1 month in Belgian nursing homes.^12^

Alongside continence promotion and appropriate use of pads and appliances,^13 14^ effective prevention and treatment includes skin cleansing and application of skin protectants.^1 15 16^ Skin protectant products comprise different formulations and ingredients such as petrolatum, zinc oxide, dimethicone or other film-forming molecules.^1 17^ Cleansers contain surface active agents, which help to dissolve the fat-soluble components in water.^18^ The odds of developing IAD if preventative measures are used are almost half (46%) the odds if they are not^11^ and when skincare regimes are implemented, costs might be reduced.^19 20^ The products and procedures for both prevention and treatment are similar^1 17^ and there are numerous options on the market, including prescription and ‘over the counter’ products. However, there is a lack of high-quality clinical trials meaning recommendations are based on low-quality evidence and expert opinion.^21^ Clear guidance is not easily available and public involvement has told us that the use of skin protectants in care homes is variable.

Although skin health can be improved using tailored and evidence-based skincare routines,^9^ nurses find it difficult to differentiate between IAD and other conditions such as pressure ulcers.^22 23^ Nurses are, however, knowledgeable about how IAD is caused but less able to diagnose and make evidence-based prevention and treatment decisions.^24 25^ Nurses’ ability to recognise IAD has been shown to improve through training,^23^ indicating the importance of continuous professional development for the prevention and treatment of IAD. Educational interventions to improve nurses’ management of IAD have taken place largely in hospital settings^24 26 27^ but are lacking in the community where the prevalence is high and unregistered care workers and informal carers provide skincare as well as registered nurses.

**Figure 1:**
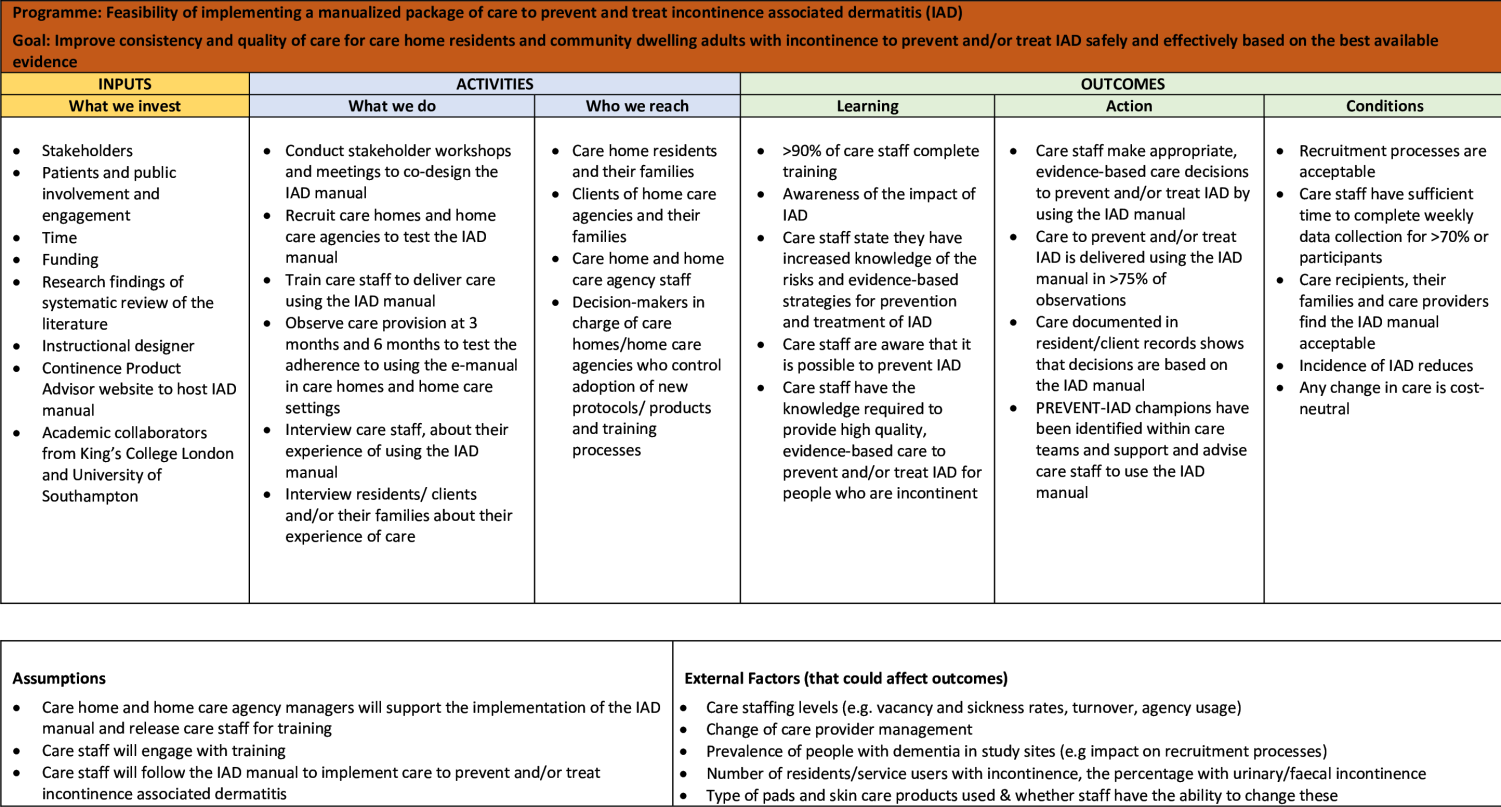
Logic model—PREVENT-IAD manual. PREVENT-IAD, PREVENTion and treatment of incontinence-associated dermatitis through a codesigned manual.

To meet the need for accessible, evidence-based clinical guidance, a manualised package of care for the prevention and treatment of IAD was codesigned with people with experience of IAD, informal carers and care staff. This intervention (to be referred to as the IAD manual in this article) is comprised of protocols in the form of an interactive treatment algorithm supported by an e-learning training programme. The IAD manual involves several interacting components (see figure 1) and as such meets the criteria for a complex intervention.^28^ Evaluation of interventions that are complex is often associated with problems of acceptability and lack of understanding of the barriers to implementation or stakeholders’ willingness to participate in research (Craig *et al*). To ensure full consideration of contextual factors, the Medical Research Council framework for the design and evaluation of complex interventions will be followed.^28 29^ The IAD manual is underpinned by a previous Cochrane review^1^ and an international consensus statement.^17^ The Cochrane review, which has been updated and is currently undergoing peer review, supports a structured skincare regime for the prevention and treatment of IAD. It includes two key steps: (1) skin cleansing to remove urine/faeces and (2) application of a skin protectant/leave-on product to avoid or minimise exposure to moisture and irritants.^16 30^ No-rinse or pH neutral skin cleansers or incontinence wipes are recommended, and traditional soap should be avoided.^1^ Although structured skincare algorithms have been proposed,^10^ this is the first specifically focusing on IAD prevention and treatment in community and social care settings.

### Objectives

The overall aim of this study is to evaluate the feasibility of implementing an IAD manual in community and social care settings.

The primary objectives are to:

- Determine the number of adults who are eligible and agree to participate.
- Assess the recruitment procedures at study sites.
- Determine retention and attrition rates for intervention and control arms of the study.
- Assess the relevance and acceptability of the intervention, data collection methods and outcome measures.
- Evaluate intervention fidelity.

Secondary objectives are to:

- Assess the incidence of IAD, IAD-related pain; satisfaction with care for the prevention and treatment of IAD and anxiety and depression.

## Methods and analysis

### Trial design and setting

A two-arm feasibility cluster randomised controlled trial (RCT) will be conducted for 6 months. It will take place in four care homes (two in region A and two in region B) and two home care agencies (one in region A and one in region B). Region A comprises the largely urban setting of London, UK and region B denotes a more rural area and relatively smaller cities of Hampshire, UK. The term, care home, describes both nursing and residential care homes where people live to receive support with personal and social care. Home care agencies are professional organisations that provide personal and social care to adults living in their own homes. These are settings and services most likely to provide care to people with incontinence.

### Eligibility criteria

- Care receivers will meet inclusion criteria if they have: (1) urinary and/or faecal incontinence with or without IAD and are residents of a care home (providing nursing and/or residential care) or (2) have with urinary/faecal incontinence with or without IAD and are receiving care from a home care agency and are (3) capable of giving valid consent, that is, voluntary and informed, or declaration by personal or nominated consultee where they lack capacity to give informed consent as defined by the UK Mental Capacity Act 2005.^31^ The UK Mental Capacity Act (2005) states an individual needs to understand the research procedures and be able to decide if they want to take part, to be assessed as having capacity to consent. If capacity to consent is unclear, clinical members of the research team will seek the opinion of family members and care managers to ascertain capacity of the potential participant. Care home residents and people living at home who receive care and are continent of both urine and faeces will not be eligible.
- Named relatives or significant others of care home residents and people receiving care from a home care agency who have incontinence, with or without IAD, will be eligible to take part in the qualitative interviews.
- Care staff who provide incontinence care for people in care homes or their own home (ie, registered nurses, care assistants) will be eligible to participate in the study. Care managers will also be invited to take part to provide information about the trial methodology and the intervention. Other personnel employed at the care home who are undertaking work experience, volunteers or short-term agency staff or are not involved in direct continence care will not be considered eligible.

### Public and patient involvement

People living with IAD and informal carers have been involved in the development of the PREVENT IAD manual (intervention) and design of the feasibility study. The study’s patient and public involvement (PPI) panel comprises three members, including a PPI coapplicant who has experience of IAD and two informal carers of family members with IAD. They have been centrally involved in determining study objectives and design. There are also two representatives from Bladder Health UK who are on the Project Steering Group. Specific PPI contributions are detailed in the relevant sections of the article below.

### Interventions

#### Intervention development

Focus groups were held with key stakeholders comprising people at risk or living with IAD (n=3) with their informal carers (n=2) and care staff (=15) in April 2021. The aim was to understand the challenges affecting the prevention and treatment of IAD and identify useful content for a care guide and best practice for implementation. Thematic analysis of the qualitative data conducted by the researchers and the PPI panel resulted in five themes illustrating uncertainty about identifying IAD; a lack of resources and evidence-based treatments; the need to improve care; the importance of good leadership to support good care and the psychological impact of IAD. The same stakeholders who participated in the focus groups provided detailed feedback on draft versions of the IAD manual via email until consensus was reached over three face-to-face workshops between March 2021 and October 2022. Changes were made to the content based on this feedback including the use of lay terms such as pee and poo and not medical terminology (eg, urine and faeces). Workshop attendees also advocated for a guide that was simple, online and interactive. They also called for posters and flyers of the online content to be available in care homes and people’s homes to enable care staff, people living with IAD and informal carers to access the information easily. The PPI panel also felt it was important to include content on the emotional and psychological impact of IAD. An audio recording about living with IAD was made by a member of the PPI panel and added to the IAD manual.

#### Intervention content

The IAD manual is an online, interactive, educational tool with asynchronous written, audio and pictorial content. The intervention includes an illustrated flowchart with six interconnected boxes; clicking on each box reveals evidence-based content about experiences of and preventing and managing IAD. A brief overview of the content of each box and corresponding learning objectives are shown in table 1. There is also a survey at the end of the IAD manual with questions based on the content of and experience of using the IAD manual. Care staff will be emailed a certificate on completion of the questions indicating which ones they answered correctly and incorrectly. They can repeat the self-test questions if they want to improve their score. Researchers will conduct a training need analysis of care staff to identify the starting point of staff knowledge about IAD. Ten care staff at each site will be trained on preventing and treating IAD following the guidelines in the IAD manual. These staff will act as the IAD champions at each site who will be asked to subsequently support their colleagues through the e-learning. The training will be face to face or online and will take up to 4 hours per member of staff. Completion of the manual will go towards staff’s continuing professional development. A training log will be used record staff attendance, number of sessions and duration of each training session by study site.

**Table 1:** Content of the intervention

Box titles	Learning outcomes
1. Improving knowledge about IAD	Describe what incontinence-associated dermatitis (IAD) is and who is most at risk Explain some of the common causes of incontinence of pee and/or poo Describe how incontinence of pee and/or poo affects people and their families
2. Assessment and management of incontinence and promoting continence	Describe what should happen when a person with incontinence of pee or poo is assessed by a nurse Explain how to take samples of pee or poo if asked Explain how to support a person to follow a toileting programme Explain what to do and what not to do when using incontinence pads
3. Steps to prevent skin damage	Describe which skin cleansers to use and which not to use Describe which products can be left on the skin to protect against IAD
4. Identifying IAD	Describe what changes are seen in the skin when IAD develops Explain how to assess the skin when cleansing or changing continence products.
5. Seeking treatment advice	Understand that skin may be damaged by other causes Know when to ask for advice and who to ask
6. Managing IAD	Describe how to treat skin which has damage caused by pee and/or poo (IAD) Explain the key steps to prevent the damage from getting worse and help skin to heal. Know when to seek help

#### Control sites

Care will remain the same during the trial for those care homes or home care agencies that have been allocated to provide usual care to prevent or treat IAD for the recruited residents and those living at home. They will have no additional training and no access to the IAD Manual.

### Recruitment

Our PPI panel and research team networks will advise on cluster sites to invite into the study. Forty-eight people with urinary and/or faecal incontinence (with or without IAD) will be recruited per study site to take part in the feasibility RCT. Researchers will liaise with care staff managers at each study site and ask them to identify potential participants they know are incontinent. If someone is not capable of deciding if they want to take part, a personal consultee will be sought (ie, nominated next of kin or whoever is nominated as the first point of contact in the person’s records). If it is not possible to either identify or contact that person within 2 weeks, then a nominated consultee independent of the project such as the person’s GP or nominated care manager will be approached. If no response from the nominated consultee is received after 2 weeks, then the person will not be included in the study. If a personal consultee is identified after a nominated consultee has signed a declaration, then the participant will be reconsented under the personal consultee process.

### Sample size

**Figure 2:**
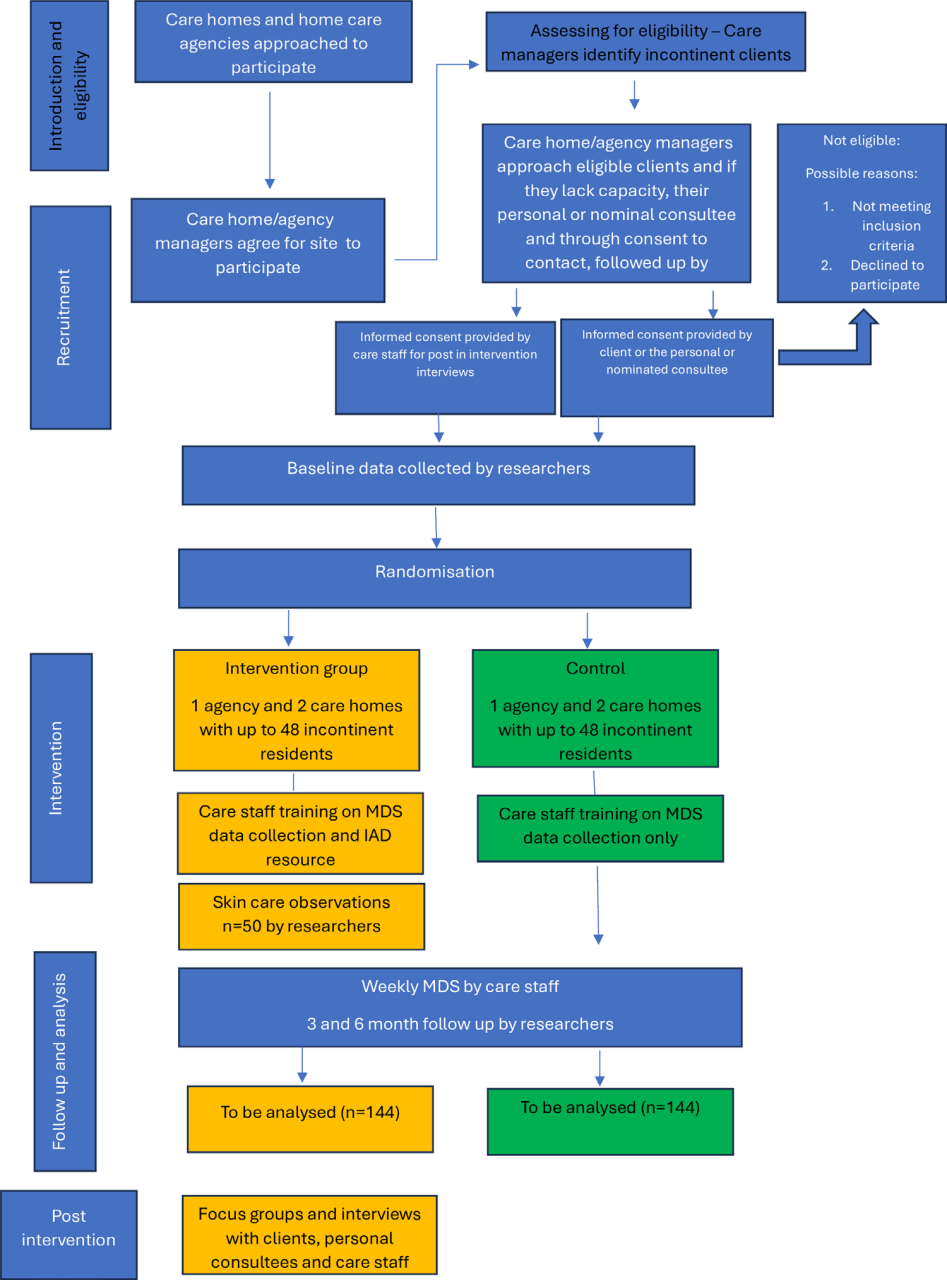
Participant flow. IAD, incontinence‐associated dermatitis; MDS, Minimum Data Set.

Two care agencies and four large care homes each with approximately 100 residential and nursing care beds will be recruited and randomised as a cluster (one home care agency and two care homes in region A, with equivalents in region B). An IAD prevalence of 30% would provide 180 people with IAD (from 600) and would be sufficient to estimate recruitment and retention rates (80% recruitment, n=144) with a maximum margin of error of approximately ±7%. Forty-eight individual participants with incontinence (with or without IAD) will be recruited per site, anticipating a mean of 58 people with incontinence per 100 beds, and 30 with IAD per 100 beds.^7 12^ An overview of the planned participant flow is shown in figure 2.

### Sequence allocation, randomisation, allocation concealment and blinding

A computer-generated randomisation sequence with 1:1 allocation to either implement the IAD care manual or usual care (control) to prevent and treat IAD will be used. The sites will be stratified to ensure balance between arms in home care agency and care homes (one home care agency and two care homes in each arm). Staff at the six study sites cannot be blinded as they will know whether they are using the IAD manual or providing usual care to prevent and treat IAD. Participants will be blinded as they will not know whether they are receiving care from the IAD manual. Care staff will be asked not to disclose the use of the manual to the participants whom they treat. The trial statistician will not be blinded, but the senior statistician will remain blinded throughout the study. The research team members who are consenting, delivering the training and conducting data collection will be unblinded. All other research team members will remain blind to the allocation.

### Data collection methods

#### Process outcomes

A record of recruitment and retention rates (cluster and individual), attrition and completion of data collection by staff will be completed by researchers. The logic model (figure 1) will be used to monitor intervention fidelity and provide insight into how the intervention did or did not work in practice, identify any unintended consequences and refine the design of the definitive trial. A list of key assumptions and uncertainties will be explored by non-participant observation for 15–30 hours per care home and home care agency (at different times from early morning to evening). The aim is to observe at least 50 skincare procedures at each site at 3 and 6 months to assess adherence to the IAD Manual in a process evaluation. The number of non-participant observations is based on a pragmatic decision as there is little guidance as to the number of observations required to capture a complex intervention.^32^ Qualitative data from the observations will be collected at 3 months and then at 6 months during the feasibility study by the researchers.

Acceptability of the intervention at the three intervention study sites and acceptability of the study design for people living with incontinence and family members/significant others from all six study sites will be explored in interviews. A purposive sample of at least eight people living with incontinence who have capacity to consent and participate, from each of the three study sites that delivered the IAD manual will be invited to take part in individual or paired (with their family member/significant other) interview to understand their views of receiving care based on the IAD manual. Personal or nominated consultees of care home residents and adults living at home who lack capacity to consent will also be invited to participate in the interviews, so that these participants are not unfairly excluded. A purposive sample of at least eight care staff from each of the intervention sites will also be invited to participate in a focus group or an individual interview if they are unable to attend the focus groups. This is to explore if the intervention was used as intended, how it worked or did not work in practice, adherence to the IAD manual, and its acceptability among those delivering care. All interviews (or focus groups) will take place at the end of the 6-month feasibility trial. Staff participants will be purposively sampled for interview to reflect a range of genders and care roles, identified IAD champions and length of experience. See onlinesupplemental files 1^ 2^.

Care recipients will be purposively sampled to ensure interviews and observations of the IAD manual use take place across a range of participants to reflect a range of different (1) genders; (2) ages; (3) skin tones; (4) BMI (under/overweight/obese); (5) with IAD or intact skin; (6) with urinary incontinence, faecal incontinence or both.

#### Clinical outcomes

A member of the research team, who is a qualified health professional, will conduct a skin examination of the participants where moisture resulting from incontinence is likely to occur (buttocks region). This skin examination will determine the prevalence and severity of IAD indicated by the appearance of erythema, erosion, maceration and the presence of IAD pain. Participants will also be asked about their satisfaction with care and their mental health. These data will be collected at the start of the feasibility cluster RCT, at 3 months and at 6 months using the following outcome measures:

1. Ghent Global IAD Categorization Tool (GLOBIAD).^33^
2. Minimum Data Set (MDS) for IAD.^2^
3. Incontinence-Associated Dermatitis Intervention Tool (to enable assessment of darker skin tones).^34^
4. Wong-Baker FACES Pain Rating Scale.^35^
5. The Short Assessment Patient Satisfaction (SAPS).^36^
6. Hospital Anxiety and Depression Scale (requested by PPI panel to include of assessment of psychological impacts of IAD)^37^

Care staff from all six study sites will also use the MDS for IAD to collect weekly data from the recruited adults living in care homes and their own homes. Therefore, the MDS will be completed on a single day concurrently but independently by a clinical member of the research team (at baseline, 3 months and 6 months) and the recruited care staff (weekly) across all study sites to assess the prevalence and severity of IAD if present. The quantitative data from these data collection tools will be collected using the online survey tool: https://www.onlinesurveys.ac.uk/, which is UK general data protection regulation (GDPR) compliant and certified to ISO 27001 standard. See onlinesupplemental files 3^ 4^.

### Training staff for data collection

Ten care staff at each study site will be provided with training on data collection from the research team. Researchers will enquire if the care staff already have experience of inputting data into online systems such as incident reports. This will enable researchers to pitch the training according to level(s) of staff digital literacy. Care staff will collect the data on IAD using either the paper versions or online survey tool. See schedule of activities in table 2.

**Table 2:** Schedule of enrolment, measurement time points, interventions, assessments and variables

	Enrolment	Baseline	Allocation	Week 12	Week 24	Post
Eligibility	X					
Informed consent	X					
**Randomisation and allocation**			x			
**Participant characteristics**						
Demographic variables (age, sex, ethnicity)		x		x	x	
Care setting		x		x	x	
**Clinical outcomes**						
Ghent Global IAD Categorization Tool		x		X	X	
Minimum Data Set (MDS) for IAD		x		X	X	
Incontinence-Associated Dermatitis Intervention Tool		x		X	X	
Wong-Baker FACES Pain Rating Scale		x		X	X	
The Short Assessment Patient Satisfaction		x		X	x	
Hospital Anxiety and Depression Scale		x		X	x	
Care staff completion of MDS				Once a week for 24 weeks		
**Feasibility outcomes**						
Recruitment and retention rates (cluster and individual)		x		x	X	
Attrition		x		x	x	
Intervention fidelity (non-participant observations of skincare)				x	X	
Completion of data collection (by staff)				x	x	
Acceptability of the intervention (interviews with clients and staff)						X
**Interventions and study-related procedures**						
Training of staff to complete the minimum data set		X	X			
Training of staff in the intervention site only in the IAD manual			X			

IADincontinence‐associated dermatitisMDSMinimum Data Set

### Data analysis

Quantitative data will be sent to the trial statistician for data cleaning. The data will be extracted in csv format and will be imported into Stata or R statistical package for data analysis. The baseline characteristics and outcome data will be described by treatment arm using descriptive statistics. No significance testing will be carried out.

### Feasibility outcome analysis

The analysis will estimate rates and proportions relating to each of the feasibility outcomes, recruitment and attrition rate, of the study. The proportions will be produced alongside the 95% CI for that proportion and the raw count which those proportions are based on. The recruitment of participants over time will be presented in a plot (number or participants vs time). The recruitment into the trial will be summarised in a Consolidated Standards of Reporting Trials diagram.^38^

### Pilot outcome analysis

The analysis of GLOBIAD and MDS, Wong-Baker FACES Pain Rating Scale will estimate proportions of patients and 95% CI. The outcomes measured using the Incontinence-Associated Dermatitis Intervention Tool, the SAPS questionnaire, the Hospital Anxiety and Depression Scale will be presented as mean and SD for each treatment group. The analysis will be carried out on the trial population using an intention to treat analysis. Completeness of the data and indication of difference will be assessed to inform selection of feasible clinical outcomes for a definitive trial. There will be no imputation to deal with missing data.

### Subgroup analyses

Subgroup analysis using summary statistics will be based on type of incontinence (urinary/faecal/both), gender and ethnicity.

### Prevalence and incidence

Prevalence, incidence and SD of IAD will be estimated to inform sample size calculations for a definitive RCT following the below thresholds for feasibility:

- ≥3 care homes/care agencies (50% of those participating) with ≥10% IAD prevalence before the intervention.
- Recruitment by care home or home care agency staff—70% of patients/residents are screened against inclusion/exclusion criteria.
- 70% completion of outcome measures by care staff.

### Process evaluation

Thematic analysis of qualitative data will be undertaken inductively^39^ to explore in-depth understanding of the functioning of the intervention and proposed methods, that is, how they are experienced by recipients and staff, which may affect feasibility of the intervention and a future trial design. Clusters’ willingness to be randomised; the reasons for attrition; and if the IAD Manual and training plan were acceptable to interviewees will also be reviewed. Fidelity will be confirmed if the processes specified in the IAD manual were followed in at least 75% of observations.

### Data management

Personal identifiable data will be stored by each university in region A and region B about the recruited participants. Signed paper consent forms will be scanned by researchers and then emailed to the researcher’s email account using secure end to end encryption. The data will be kept for 25 years as the study might involve participants who lack capacity to consent according to the Sponsor’s policy.

### Monitoring

The sponsor, through the chief investigator (SW) and mentors (CN and RH) will serve as monitors for this feasibility trial. A Trial Management Group (TMG) will be convened comprising the chief investigator, mentors, coapplicants (MF, PW), the PPI coapplicant (CC) and international collaborators (DB, JK). The TMG will determine the appropriate extent and nature of monitoring based on considerations such as the objective, purpose, design, complexity and endpoints of the feasibility study. There will be on-site monitoring, before, during and after the feasibility study. An independent steering group will meet at least annually, comprising a chair, two PPI members, two health professionals with expertise in related fields and statistician, all independent of the research team, study sites and institutions involved.

### Payment

Research participants will not receive any payments, reimbursement of expenses or any other benefits or incentives for taking part in the RCT. Funding will be given to each intervention site to release care staff for training in the use of IAD manual including the training/education programme (n=10 staff per site, for 4 hours per member of staff). Each participating site will be provided with funding to release of care home/agency staff for training in data collection (n=10 staff per site at all six sites) for 2 hours per member of staff.

### Harms and safeguarding

It is not anticipated that there will be any risks associated with the care staff at the three intervention sites using the IAD manual in the RCT. Recommended products and procedures to prevent and treat IAD included in the IAD manual are widely used and licensed for the intended use.^1 17^ Care staff at the intervention sites will be shown by the research team how to use the IAD manual and will be expected to follow the guidance, the warnings and precautions stipulated by the manufacturers in the information leaflets for the CE marked products they decide to use. Should a serious adverse event or adverse reaction occur due to using any of the products and/or carrying out the procedures to cleanse and protect the skin during the study, staff will be asked to report it to their manager and the research team who will ensure that a prompt assessment is completed and inform the ethics and steering committees. If a researcher has a concern that the safety and/or rights of a resident or staff member participating in the research are not being maintained, the researcher will discuss this with the chief investigator who will report this to the person in charge of the care home. Researchers who are nurses will follow professional codes of conduct if they suspect there is a risk to safety, or a vulnerable person is at risk.

### Ethics and dissemination

The trial has been approved by the Queens Square Research Ethics Committee (REC) Health Research Authority 23/LO/036, IRAS Project ID 296167. If any substantial amendments are required, these will be submitted for review by the REC will not be implemented until approved. The chief investigator and sponsor will oversee that the protocol for the feasibility cluster RCT is followed. The sponsor will be notified immediately of any case of any breaches to the protocol have occurred during the trial conduct phase.

### Consent

All participants will be given detailed study information (including using an accessible version of the information sheet for people with mild/moderate cognitive impairment). Written consent will be taken by a member of the research team who are clinicians (online supplemental file 5). In the case of care recipients lacking capacity to consent, guidance laid out in the UK Mental Capacity Act 2005^31^ will be followed, and researchers will seek an opinion from the person’s representative on what they think their relative’s/person they represent wishes would be to participation, which will be documented. All participants will have the right to withdraw from the study at any time without affecting their care. Informed consent will be an ongoing process, repeated at each encounter, to ensure continued consent and good practice.^40 41^

### Data protection and patient confidentiality

Once written informed consent or declaration (in case of adults lacking capacity to provide valid informed consent) is obtained, a unique study ID will be allocated, under which all study data will be anonymised. Identifiable participant information will be linked to participants’ study ID, stored in a password-protected database only accessible to those members of the research team whose role requires access to personal identifiers. The chief investigator, the coapplicant overseeing the recruitment for region B (PW) and the statisticians (FF) will have access to the final trial dataset.

All research data will be password protected and held separately to identifiable information, on a password-protected database. Signed paper consent forms will be scanned by researchers and then emailed to the research team using secure end to end encryption. The electronic versions will be stored by the researchers on a password-protected software (university purchased software) linked database. All electronic data will be encrypted. The paper copies will be kept by the study sites. The investigators and study site staff will comply with the requirements of the Data Protection Act 2018. All information related to study participants will be kept confidential and managed in accordance with General Data Protection Regulation, the Research Governance Framework for Health and Social Care and Research Ethics Committee Approval. All research data will be stored on a secure password-protected computer under the study ID. All paper copies of study data will be stored under ID number and kept in locked offices within the research facilities; research data will be held separately to identifiable information. No identifiable data will be included in research publications or progress reports.

### Dissemination

Following completion of the study, the feasibility trial results will be published on the funders website. The results will be uploaded to the ISRCTN registry (ISRCTN70866724). Papers will be prepared for national and international conferences and open access, peer-reviewed journals. The results will guide the design of a definitive cluster RCT to test the clinical and cost-effectiveness of the IAD Manual. The IAD Manual will not be disseminated until after the completion of the definitive RCT, so as not to prejudice the results. The definitive RCT protocol will be submitted for publication and registered on the ISRCTN registry. The feasibility trial results will also be disseminated to the Registered Nursing Home Association, Care England and The UK Homecare Association. Our PPI coapplicant (CC) will lead on the production of targeted outputs for inclusion in patient organisation (eg, Promocon; Bladder Health UK) newsletters and social media channels.

## Discussion

This feasibility trial will provide data on recruitment and attrition rates, acceptability of the IAD manual, intervention fidelity and clinical outcome data on IAD. This will enable the research team to determine whether a definitive cluster RCT of the intervention (IAD manual) can be conducted to test the clinical and cost-effectiveness of the IAD manual.

Implementation of a manualised package of care for IAD prevention and treatment will offer a potentially scalable intervention to guide evidence-based practice across a diverse range of settings and professional groups as well as for patients and informal carers. Currently, standard care for IAD is delivered by care organisations in accordance with their local procedures. Therefore, care can be variable with a wide range of products used in without a clinical guidance and little evidence of their effectiveness. Successful implementation of the IAD manual could lead to improved skills of care staff in the recognition, prevention and management of IAD through the appropriate use of products and skincare regimes. Communication and information sharing between care homes, community care agencies and hospital settings could also be improved, resulting in efficiency savings. People living with incontinence would potentially receive more appropriate care to prevent and treat IAD and inappropriate care that could worsen IAD (eg, cleansing with traditional soap and water) could be reduced. The incidence of IAD in care homes and other community settings could also be reduced. A clinically and cost-effective intervention to prevent and treat IAD, such as the IAD manual, could also lead to savings through reduction in the incidence of pressure ulcers and inappropriate prescribing/product selection.

One of the most pressing challenges to the adoption of the findings from this and a future trial of the IAD Manual lies in translating the evidence through the online resource to staff from a diverse care home and home care agency sector. Some care home/home care agency staff may have low literacy skills and there is often a high turnover of staff in metropolitan areas. There are also time pressures and restricted budgets in the social care environment, which makes training difficult to implement.^42 43^ The process evaluation will enable us to understand these issues and how best they may be overcome in definitive trial, such as producing a pictorial ‘quick guide’ synopsis. Implementation of the IAD manual may also be compromised by the availability of products in primary and secondary care. Stakeholders will be asked to identify the most used, available and affordable products across care settings and simplify recommendations, enabling implementation widely. Engaging with key stakeholders in this way enables a ‘real-world implementation’ of a complex intervention.^28 44^

## supplementary material

10.1136/bmjopen-2024-092338 online supplemental file 1

10.1136/bmjopen-2024-092338 online supplemental file 2

10.1136/bmjopen-2024-092338 online supplemental file 3

10.1136/bmjopen-2024-092338 online supplemental file 4

10.1136/bmjopen-2024-092338 online supplemental file 5
