# Supplementary material for: PREVENTion and treatment of incontinence-associated dermatitis through a codesigned manual (PREVENT-IAD): a study protocol for a feasibility cluster randomised controlled trial with a nested process evaluation
Source: BMJ Open. 2024 Dec 23;14(12):e092338. doi: 10.1136/bmjopen-2024-092338 (PMC11667359; doi:10.1136/bmjopen-2024-092338)
Supplement: online supplemental file 1 [file bmjopen-14-12-s001.docx]

# PREVENT-IAD Topic guide for individual interviews with residents/family members/care recipients

The aim is to find out from the residents/community dwelling adults receiving incontinence care and their family members (if paired interviews take place), and from personal or nominated consultees about the care provided from the IAD care package.

- Can you please tell us about your/your family member’s experience of living with incontinence?
  - How long have you/your family member been experiencing incontinence symptoms?
- What impact has this had on you/your family?
  - How has this affected your/your family member’s skin?
  - Have you/your family member had any skin problems and how did this affect you/your family member?
- Before we trained your/your family member’s care staff in using the IAD care package how was your/your family member’s skin care managed?
  - How was your/your family member’s skin cleansed for daily hygiene and after an episode of incontinence?
  - What products were applied to your/your family member’s skin?
  - How satisfied were you/your family member with this care?
- What has been your experience of incontinence skin care since your/your family member’s care staff were trained in using the IAD care package?
  - How has your/your family member’s care changed?
  - How has this impacted on the condition of your/your family member’s skin?
  - What do you think the physical and emotional benefits and challenges were for you/your family member’s care staff in using this care package?
  - How satisfied were you/your family member with this care?
  - How much were you able to follow the guidance in the IAD care package to care for your/your family member’s skin?
  - What did you think/how did you feel about the language/terminology in the IAD care package?
  - Is there anything you would recommend to improve the IAD care package?
