# Supplementary material for: PREVENTion and treatment of incontinence-associated dermatitis through a codesigned manual (PREVENT-IAD): a study protocol for a feasibility cluster randomised controlled trial with a nested process evaluation
Source: BMJ Open. 2024 Dec 23;14(12):e092338. doi: 10.1136/bmjopen-2024-092338 (PMC11667359; doi:10.1136/bmjopen-2024-092338)
Supplement: online supplemental file 2 [file bmjopen-14-12-s002.docx]

# PREVENT-IAD Topic guide for focus group/individual interviews with care staff

The aim is to find out from care staff how using the IAD care package fit with their existing workload, how this intervention did or did not work in practice and identify any unintended consequences.

- Can you please tell us about your experience of caring for people who are incontinent?
  - How long have you been caring for people who are incontinent?
  - How do feel about caring for people who are incontinent?
  - How much training had you had previously in providing care for people who are incontinent?
- Before we introduced the IAD care package how was skin care provided to people who are incontinent?
  - How much training had you had previously in providing **skin care** to people who are incontinent?
- What was your experience of using the IAD care package?
  - How and where did you access the IAD care package? (e.g. on a mobile phone/device computer; in an office/staff room/ resident’s place of care/at home)
  - How often did you access/refer to the IAD care package (flow chart or e-learning)?
  - How did you use the IAD care package to make decisions about the care you would provide?
  - How did this support/change how you managed skin care for people who are incontinent and at risk of IAD?
  - How did this support/change how you treated the skin of people who developed IAD?
  - How did using the IAD care package fit with your existing workload?
  - What impact has using the IAD care package had on your practice?
  - What were the benefits to you of using the IAD care package?
  - Did you encounter any challenges in using the IAD care package? What were these?
  - How do you communicate a person’s IAD status and skin care regime to secondary care?
  - How could this communication with secondary care be improved?
  - What did you think/how did you feel about the language/terminology in the IAD care package?
  - Is there anything you would recommend to improve the IAD care package?
