# Supplementary material for: PREVENTion and treatment of incontinence-associated dermatitis through a codesigned manual (PREVENT-IAD): a study protocol for a feasibility cluster randomised controlled trial with a nested process evaluation
Source: BMJ Open. 2024 Dec 23;14(12):e092338. doi: 10.1136/bmjopen-2024-092338 (PMC11667359; doi:10.1136/bmjopen-2024-092338)
Supplement: online supplemental file 3 [file bmjopen-14-12-s003.pdf]

# PREVENT-IAD feasibility study phase 3: data collection tools for researchers

## PREVENT-IAD feasibility study phase 3: data collection tools for researchers to complete

Collection of data at the start, 3 months and 6 months of the study

To the research team for the PREVENT-IAD study being carried out by King's College London and the University of Southampton. IAD stands for Incontinence Associated Dermatitis and is skin damage caused by pee and poo remaining on the skin for a long time. In the first two phases of the PREVENT-IAD project, we developed the Incontinence Associated Dermatitis (IAD) Manual/package of care. The Manual is comprised of an IAD skin care flow chart/set of rules and an e-learning programme to guide the prevention and treatment of IAD. In phase 3 of the PREVENT-IAD project, we would like you to collect some data when you accompany the care staff who provide skin care for incontinence/IAD to the care home residents or to the adults living at home. These participants have agreed to take part in the PREVENT-IAD study and for you and the care staff to assess their skin condition as a result of the care they are receiving. We will be asking you to complete these six questionnaires at the start of the trial, then at 3 months and then at the end of the trial at 6 months. By completing the six questionnaires at these three different time points, you will provide us with information that will tell us if we are collecting the right type of data to assess the prevention and treatment for IAD. We will also find out how the participants are feeling and if they are satisfied with their care. We will start by asking you to record the participants' identification number and non-identifiable information. We will then ask you to complete the following data collection tools: Ghent Global IAD Categorisation Tool (GLOBIAD); Minimum Data Set (MDS) for IAD; Incontinence-Associated Dermatitis Intervention Tool; Wong-Baker FACES Pain Rating Scale; The Short Assessment Patient Satisfaction (SAPS) questionnaire; The Hospital Anxiety and Depression Scale We thank you for your participation in this study and for collecting the data.

## Participants' identification number and non-identifiable information: Collection of data at the start, 3 months and 6 months of the study

### 1. Study ID number *

### 2. Data collection phase *

- ◯ Baseline
- ◯ 3 month
- ◯ 6 month

### 3. Year of birth *

### 4. Gender *

- ◯ Male
- ◯ Female

### 5. Setting *

- ☐ Care home
- ☐ Home care agency

## Ghent Global IAD Categorisation Tool (GLOBIAD): Collection of data at the start, 3 months and 6 months of the study

The Ghent Global IAD Categorisation tool (GLOBIAD) sets out the categories for IAD. This involves the user of the tool looking at the affected skin areas of the person they are caring for and deciding which of the four categories are applicable for that person. Please can you look at the four categories for IAD and the questions set out below. Note: the IAD categories do not necessarily relate to the natural history of IAD and are not intended to suggest how IAD may develop or progress. However the tool may be helpful to record IAD.

### 6. Does the person have any one of the four GLOBIAD categories of IAD? Please see below for the categories with the images. *

☐ Yes

☐ No

### 7. If you answered yes that the person has IAD, please select which one of the four GLOBIAD categories that is applicable to that person.

○ Category 1A: Persistent redness without clinical signs of infection

○ Category 1B: Persistent redness with clinical signs of infection

○ Category 2A: Skin loss without clinical signs of infection

○ Category 2B: Skin loss with clinical signs of infection

Category 1A: Persistent redness without clinical signs of infection

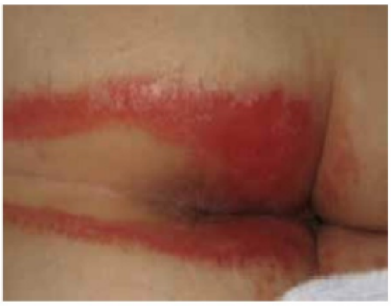

Critical criteria: Persistent redness: a variety of tones of redness may be present. Patients with darker skin tones, the skin may be paler or darker than normal, or purple in colour.

Additional criteria • Marked areas or discolouration from a previous (healed) skin defect • Shiny appearance of the skin • Macerated skin • Intact vesicles and/or bullae • Skin may feel tense or swollen at palpation • Burning, tingling, itching or pain

Category 1B: Persistent redness with clinical signs of infection

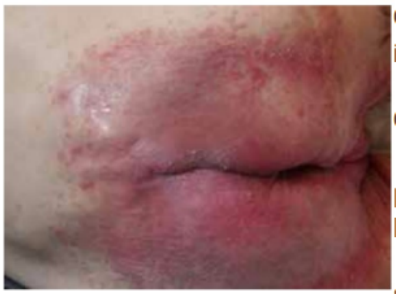

Critical criteria Persistent redness: a variety of tones of redness may be present. Patients with darker skin tones, the skin may be paler or darker than normal, or purple in colour. Signs of infection: such as white scaling of the skin (suggesting a fungal infection) or satellite lesions (pustules surrounding the lesion, suggesting a Candida albicans fungal infection).

Additional criteria • Marked areas or discolouration from a previous (healed) skin defect • Shiny appearance of the skin • Macerated skin • Intact vesicles and/or bullae • Skin may feel tense or swollen at palpation • Burning, tingling, itching or pain

Category 2A: Skin loss without clinical signs of infection

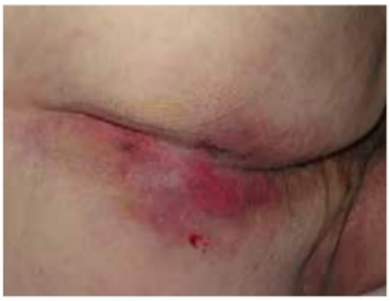

Critical criteria Skin loss: skin loss may present as skin erosion (may result from damaged/eroded vesicles or bullae), denudation or excoriation. The skin damage pattern may be diffuse.

Additional criteria • Persistent redness: a variety of tones of redness may be present. Patients with darker skin tones, the skin may be paler or darker than normal, or purple in colour • Marked areas or discolouration from a previous (healed) skin defect • Shiny appearance of the skin • Macerated skin • Intact vesicles and/or bullae • Skin may feel tense or swollen at palpation • Burning, tingling, itching or pain

Category 2B: Skin loss with clinical signs of infection

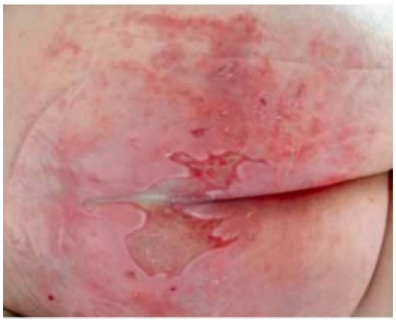

Critical criteria Skin loss: skin loss may present as skin erosion (may result from damaged/eroded vesicles or bullae), denudation or excoriation. The skin damage pattern may be diffuse.

Signs of infection: such as white scaling of the skin (suggesting a fungal infection) or satellite lesions (pustules surrounding the lesion, suggesting a Candida albicans fungal infection), slough visible in the wound bed (yellow/brown/greyish), green appearance within the wound bed (suggesting a bacterial infection with Pseudomonas aeruginosa), excessive exudate levels, purulent exudate (pus) or a shiny appearance of the wound bed.

Additional criteria • Persistent redness: a variety of tones of redness may be present. Patients with darker skin tones, the skin may be paler or darker than normal, or purple in colour • Marked areas or discolouration from a previous (healed) skin defect • Shiny appearance of the skin • Macerated skin • Intact vesicles and/or bullae • Skin may feel tense or swollen at palpation • Burning, tingling, itching or pain

## Minimum Data Set (MDS) for IAD: Collection of data at the start, 3 months and 6 months of the study

The Minimum Data Set for Incontinence-Associated Dermatitis (IAD) provides information on the rate of IAD amongst people with incontinence (bladder or bowel leakage). Please can you ask the staff member and/or observe answers to the following questions about the person receiving care.

**8. Please select which one of the following options regarding bladder (pee) leakage applies to the person you are caring for:** *

- [ ] Not incontinent
- [ ] Occasionally incontinent
- [ ] Frequently incontinent
- [ ] Always incontinent

**9. Please select which one of the following options regarding bowel (poo) leakage applies to person you are caring for:** *

- [ ] Not incontinent
- [ ] Occasionally incontinent
- [ ] Frequently incontinent
- [ ] Always incontinent

**10. Does the person you are caring for have diarrhoea?** *

- [ ] Yes
- [ ] No

**11. How do you cleanse the skin of the person you are caring for when he or she has a bladder (pee) or bowel (poo) leakage? Please select all the options that apply.** *

- [ ] Using toilet paper
- [ ] Water and cleanser
- [ ] Water and oil

- [ ] No-rinse skin cleansers
- [ ] Cleansing form
- [ ] Single-use disposable bathing wipes

**12. How did you decide to give this care in this way? Please pick all the options that apply. ***

- [ ] Always done it this way
- [ ] Instructed by the care home manager/home care agency/nurse to do it this way
- [ ] Prescribed by a healthcare professional
- [ ] Those were the only products available
- [ ] Followed the PREVENT-IAD flow chart (answer is only applicable if you are working in one of the care homes/home care agencies using the flow chart)
- [ ] Other reason

**13. If you selected the option, other reason please state the reason(s).**

| |
|---|
| |

**14. After cleansing the skin of the person you are caring for, do you use a leave-on product? ***

- [ ] Yes
- [ ] No

**15. How did you decide to give this care in this way? Please pick all the options that apply. ***

- [ ] Always done it this way
- [ ] Instructed by the care home manager/home care agency/nurse to do it this way
- [ ] Prescribed by a healthcare professional
- [ ] Those were the only products available
- [ ] Followed the PREVENT-IAD flow chart (answer is only applicable if you are working in one of the care homes/home care agencies using the flow chart and not at baseline)
- [ ] Other reason

**16. If you selected the option, other reason please state the reason(s).**

**17.** Do you use an anti-microbial agent (medicines used to prevent and treat infections)? *

- ☐ Yes
- ☐ Yes, on prescription
- ☐ No

**18.** How did you decide to give this care in this way? Please pick all the options that apply. *

- ☐ Always done it this way
- ☐ Instructed by the care home manager/home care agency/nurse to do it this way
- ☐ Prescribed by a healthcare professional
- ☐ Those were the only products available
- ☐ Followed the PREVENT-IAD flow chart (answer is only applicable if you are in the care home/home care agency using the flow chart)
- ☐ Other reason
- ☐ Not applicable

**19.** If you selected the option, other reason please state the reason(s).

**20.** Which incontinence products do you use for the person you are caring for? *

- ☐ Pads/briefs/liners
- ☐ Pull-up pants
- ☐ Underpads

**21.** How did you decide to give this care in this way? Please pick all the options that apply *

- ☐ Always done it this way
- ☐ Instructed by the care home manager/home care agency/nurse to do it this way
- ☐ Prescribed by a healthcare professional

- [ ] Those were the only products available
- [ ] Followed the PREVENT-IAD flow chart (answer is only applicable if you are working in one of the care homes/home care agencies using the flow chart)
- [ ] Other reason
- [ ] Not applicable

22. If you selected the option, other reason please state the reason(s).

23. Is the person you are caring for on a urinary (pee) toileting programme? *

- [ ] Yes
- [ ] No

24. If you selected yes, please provide the start date for the urinary (pee) toileting programme.

25. Is the person you are caring for on a bowel (poo) toileting programme? *

- [ ] Yes
- [ ] No

26. If you selected yes, please provide the start date bowel (poo) toileting programme.

27. Do you think the person's skin condition has changed in the last week? *

- [ ] Stayed the same as last week
- [ ] Better than last week

- [ ] Worse than last week

# Incontinence-Associated Dermatitis Intervention Tool (IADIT): Collection of data at the start, 3 months and 6 months of the study

The IADIT tool has clear pictures, definitions and short descriptions of skin changes and rashes to illustrate the risk and the stages of severity for IAD.

**28. Does the person have any one of the four IADIT categories for IAD? Please see below for the categories with the images** *

☐ Yes

☐ No

**29. If you answered yes that the person has or is at risk of IAD, please select which one of the four IADIT categories that is applicable to that person**

◯ High risk of IAD

◯ Early IAD

◯ Moderate IAD

◯ Severe IAD

HIGH-RISK (there is no image for this category)

Skin is not erythematous or warmer than nearby skin but may show scars or colour changes from previous IAD episodes and/or healed pressure ulcer(s). Person not able to adequately care for self or communicate need and is incontinent of liquid stool at least 3 times in 24 hours.1

1. Bliss DZ, Zehrer C, Savik K, et al. Incontinence-associated skin damage in nursing home residents: a secondary analysis of a prospective, multicenter study. Ostomy Wound Manage. 2006;52:46–55

**EARLY IAD**

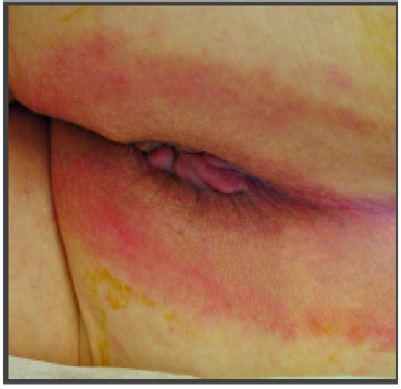

Skin exposed to stool and/or urine is dry, intact, and not blistered, but is pink or red with diffuse (not sharply defined), often irregular borders. In darker skin tones, it might be more difficult to visualise colour changes (white, yellow, very dark red/purple) and palpation may be more useful. Palpation may reveal a warmer temperature compared to skin not exposed. People with adequate sensation and the ability to communicate may complain of burning, stinging, or other pain.

**MODERATE IAD**

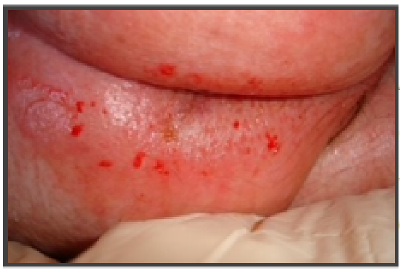

Affected skin is bright or angry red – in darker skin tones, it may appear white, yellow, or very dark red/purple. Skin usually appears shiny and moist with weeping or pinpoint areas of bleeding. Raised areas or small blisters may be noted. Small areas of skin loss (dime size) if any. This is painful whether or not the person can communicate the pain.

**SEVERE IAD**

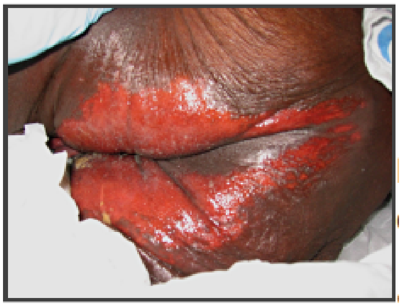

Affected skin is red with areas of denudement (partial-thickness skin loss) and oozing/bleeding. In dark-skinned persons, the skin tones may be white, yellow, or very dark red/purple. Skin layers may be stripped

off as the oozing protein is sticky and adheres to any dry surface.

## Wong-Baker FACES® Pain Rating Scale: Collection of data at the start, 3 months and 6 months of the study

The Wong-Baker FACES® Pain Rating Scale was originally created to help children communicate about their physical pain. Now the scale is used around the world with people ages 3 and older, so pain management can be addressed. The Scale is a self-assessment tool so please speak to the person you are caring for and ask them which face applies to them to determine their level of pain.

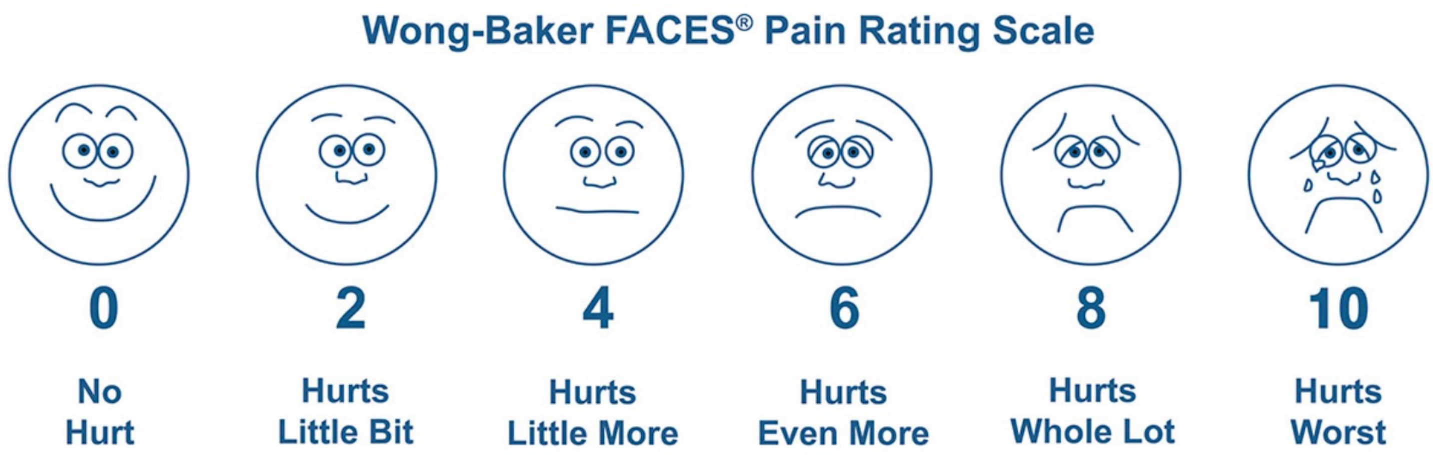


30. Please can you speak to the person and ask them which face applies to them. Please then select the answer. *

- ◯ No hurt
- ◯ Hurts Little Bit
- ◯ Hurts Little More
- ◯ Hurts Even More
- ◯ Hurts Whole Lot
- ◯ Hurts Worst
- ◯ Unable to answer

# The Short Assessment of Patient Satisfaction (SAPS) questionnaire: Collection of data at the Collection of data at the start, 3 months and 6 months of the study

The Short Assessment of Patient Satisfaction (SAPS) is a short, reliable and valid seven item scale that can be used to assess patient satisfaction with their treatment. For this study, the questionnaire is being used to assess the satisfaction of each care home resident or adults receiving care in the community with either the IAD care package if they are receiving this or with their usual care to prevent or treat IAD. Instructions: After reading each question, please can you assist the care home resident/community dwelling adult receiving care at home to select the answer that best describes their satisfaction with their care to prevent or treat IAD. Please check that all questions have been answered.

**31. How satisfied are you with the effect of your treatment/care?** *

- ☐ Very satisfied
- ☐ Satisfied
- ☐ Neither satisfied or dissatisfied
- ☐ Dissatisfied
- ☐ Very dissastified
- ☐ Unable to answer

**32. How satisfied are you with the explanations the (doctor/other health professional) has given you about the results of your treatment/care?** *

- ◯ Very dissatisfied
- ◯ Dissatisfied
- ◯ Neither satisfied or dissatisfied
- ◯ Satisfied
- ◯ Very satisfied
- ◯ Unable to answer

**33. The (doctor/other health professional) was very careful to check everything when examining you.** *

- ◯ Strongly agree

- Agree
- Not sure
- Disagree
- Strongly disagree
- Unable to answer

**34. How satisfied were you with the choices you had in decisions affecting your health care? ***

- Very dissatisfied
- Dissatisfied
- Neither satisfied or dissatisfied
- Satisfied
- Very satisfied
- Unable to answer

**35. How much time did you feel respected by the (doctor/other health professional)? ***

- All of the time
- Most of the time
- About half of the time
- Some of the time
- None of the time
- Unable to answer

**36. The time you had with the (doctor/other health professional) was too short ***

- Strongly agree
- Agree
- Not sure
- Disagree
- Strongly disagree
- Unable to answer

**37. Are you satisfied with the care you received in the (hospital/clinic)? ***

- ◯ Very satisfied
- ◯ Satisfied
- ◯ Neither satisfied or dissatisfied
- ◯ dissatisfied
- ◯ Very dissatisfied
- ◯ Unable to answer

## Hospital Anxiety and Depression Scale (HADS): Collection of data at the start, 3 months and 6 months of the study

The Hospital Anxiety and Depression Scale (HADS) measures anxiety and depression. The questionnaire comprises of seven questions for anxiety and seven questions for depression. Please can you assist the person to complete this questionnaire by selecting one of the options for each of the 14 questions. Please check that all questions have been answered.

**38. I feel tense or 'wound up':** *

- ☐ Most of the time
- ☐ A lot of the time
- ☐ From time to time, occasionally
- ☐ Not at all
- ☐ Unable to answer

**39. I still enjoy the things I used to enjoy:** *

- ☐ Definitely as much
- ☐ Not quite so much
- ☐ Only a little
- ☐ Hardly at all
- ☐ Unable to answer

**40. I get a sort of frightened feeling as if something awful is about to happen:** *

- ☐ Very definitely and quite badly
- ☐ Yes, but not too badly
- ☐ A little, but it does not worry me
- ☐ Not at all
- ☐ Unable to answer

**41. I can laugh and see the funny side of things:** *

- [ ] As much as I always could
- [ ] Not quite so much now
- [ ] Definitely not so much now
- [ ] Not at all
- [ ] Unable to answer

### 42. Worrying thoughts go through my mind: *

- [ ] A great deal of the time
- [ ] A lot of the time
- [ ] Not too often
- [ ] Very little
- [ ] Unable to answer

### 43. I feel cheerful: *

- [ ] Never
- [ ] Not often
- [ ] Sometimes
- [ ] Most of the time
- [ ] Unable to answer

### 44. I can sit at ease and feel relaxed: *

- [ ] Definitely
- [ ] Usually
- [ ] Not often
- [ ] Not at all
- [ ] Unable to answer

### 45. I feel as if I am slowed down: *

- [ ] Nearly all the time
- [ ] Very often

☐ Sometimes

☐ Not at all

☐ Unable to answer

**46. I get a sort of frightened feeling like 'butterflies' in the stomach: ***

☐ Not at all

☐ Occasionally

☐ Quite often

☐ Very often

☐ Unable to answer

**47. I have lost interest in my appearance: ***

☐ Definitely

☐ I do not take as much care as I should

☐ I may not take quite as much care

☐ I take just as much care as ever

☐ Unable to answer

**48. I feel restless as I have to be on the move: ***

☐ Very much indeed

☐ Quite a lot

☐ Not very much

☐ Not at all

☐ Unable to answer

**49. I look forward with enjoyment to things: ***

☐ As much as I ever did

☐ Rather less than I used to

☐ Definitely less than I used to

☐ Hardly at all

- [ ] Unable to answer

**50. I get sudden feelings of panic:** *

- [ ] Very often indeed
- [ ] Quite often
- [ ] Not very often
- [ ] Not at all
- [ ] Unable to answer

**51. I can enjoy a good book or radio or TV programme:** *

- [ ] Often
- [ ] Sometimes
- [ ] Not often
- [ ] Very seldom
- [ ] Unable to answer

## Additional question at the start, 3 months and 6 months of the study

52. Is there anything else you would like to tell us about the person's skin care for incontinence/IAD?
