# Supplementary material for: PREVENTion and treatment of incontinence-associated dermatitis through a codesigned manual (PREVENT-IAD): a study protocol for a feasibility cluster randomised controlled trial with a nested process evaluation
Source: BMJ Open. 2024 Dec 23;14(12):e092338. doi: 10.1136/bmjopen-2024-092338 (PMC11667359; doi:10.1136/bmjopen-2024-092338)
Supplement: online supplemental file 4 [file bmjopen-14-12-s004.pdf]

PREVENT-IAD feasibility study - phase 3: minimum data set only

PREVENT-IAD feasibility study - phase 3: minimum data set only for the care staff to complete

To be completed by care staff each week

Hello The care home/home care agency you are working at has agreed to take part in the PREVENT-IAD study which is being carried out by a team of researchers at King's College London and the University of Southampton. IAD stands for Incontinence Associated Dermatitis and is skin damage caused by pee and poo remaining on the skin for a long time. In the first two phases of the PREVENT-IAD project, we developed the Incontinence Associated Dermatitis (IAD) Manual/package of care. The Manual is comprised of an IAD skin care flow chart/set of rules and an e-learning programme to guide the prevention and treatment of IAD. In phase 3 of the PREVENT-IAD project, we would like you to collect some data when you provide skin care for incontinence/IAD to the care home residents or to the adults living at home that you look after. These participants have agreed to take part in the PREVENT-IAD study and for you to assess their skin condition as a result of the care they are receiving. We would like you to complete one questionnaire when you are providing this skin care during a six months trial. You will be providing skin care using the IAD Manual guidance if your care home/home care agency has been asked to use the Manual. Alternatively you will continue to provide the usual care to treat and prevent IAD. We will be asking you to complete this questionnaire each week for 6 months. By completing the questionnaire you will provide us with information that will tell us if we are collecting the right type of data to assess the prevention and treatment for IAD. This survey is to collect data using the following data collection tool: 1. Minimum Data Set (MDS) for IAD We thank you for your participation in this study and for collecting the data.

Participants' identification number and non-identifiable information

1. Study ID number *

2. Year of birth *

3. Gender *

- ○ Male
- ○ Female

4. Setting *

- ☐ Care home
- ☐ Home care agency

Minimum Data Set (MDS) for IAD: Collection of data each week

The Minimum Data Set for Incontinence-Associated Dermatitis (IAD) provides information on the rate of IAD amongst people with incontinence (bladder or bowel leakage). Please can you answer the following questions for the person you are caring for.

5. Please select which one of the following options regarding bladder (pee) leakage applies to the person you are caring for: *

- ☐ Not incontinent
- ☐ Occasionally incontinent
- ☐ Frequently incontinent
- ☐ Always incontinent

6. Please select which one of the following options regarding bowel (poo) leakage applies to person you are caring for: *

- ☐ Not incontinent
- ☐ Occasionally incontinent
- ☐ Frequently incontinent
- ☐ Always incontinent

7. Does the person you are caring for have diarrhoea? *

- ☐ Yes
- ☐ No

8. How do you cleanse the skin of the person you are caring for when he or she has a bladder (pee) or bowel (poo) leakage? Please select all the options that apply. *

- ☐ Using toilet paper
- ☐ Water and cleanser
- ☐ Water and oil

- [ ] No-rinse skin cleansers
- [ ] Cleansing form
- [ ] Single-use disposable bathing wipes

9. How did you decide to give this care in this way? Please pick all the options that apply. *

- [ ] Always done it this way
- [ ] Instructed by the care home manager/home care agency/nurse to do it this way
- [ ] Prescribed by a healthcare professional
- [ ] Those were the only products available
- [ ] Followed the PREVENT-IAD flow chart (answer is only applicable if you are working in one of the care homes/home care agencies using the flow chart)
- [ ] Other reason

10. If you selected the option, other reason please state the reason(s).

____

11. After cleansing the skin of the person you are caring for, do you use a leave-on product? *

- [ ] Yes
- [ ] No

12. How did you decide to give this care in this way? Please pick all the options that apply. *

- [ ] Always done it this way
- [ ] Instructed by the care home manager/home care agency/nurse to do it this way
- [ ] Prescribed by a healthcare professional
- [ ] Those were the only products available
- [ ] Followed the PREVENT-IAD flow chart (answer is only applicable if you are working in one of the care homes/home care agencies using the flow chart)
- [ ] Other reason

13. If you selected the option, other reason please state the reason(s).

14. Do you use an anti-microbial agent (medicines used to prevent and treat infections)? *

- [ ] Yes
- [ ] Yes, on prescription
- [ ] No

15. How did you decide to give this care in this way? Please pick all the options that apply. *

- [ ] Always done it this way
- [ ] Instructed by the care home manager/home care agency/nurse to do it this way
- [ ] Prescribed by a healthcare professional
- [ ] Those were the only products available
- [ ] Followed the PREVENT-IAD flow chart (answer is only applicable if you are in the care home/home care agency using the flow chart)
- [ ] Other reason
- [ ] Not applicable

16. If you selected the option, other reason please state the reason(s).

17. Which incontinence products do you use for the person you are caring for? *

- [ ] Pads/briefs/liners
- [ ] Pull-up pants
- [ ] Underpads

18. How did you decide to give this care in this way? Please pick all the options that apply *

- [ ] Always done it this way
- [ ] Instructed by the care home manager/home care agency/nurse to do it this way
- [ ] Prescribed by a healthcare professional

☐ Those were the only products available

☐ Followed the PREVENT-IAD flow chart (answer is only applicable if you are working in one of the care homes/home care agencies using the flow chart)

☐ Other reason

19. If you selected the option, other reason please state the reason(s).

20. Is the person you are caring for on a urinary (pee) toileting programme? *

☐ Yes

☐ No

21. If you selected yes, please provide the start date for the urinary (pee) toileting programme.

22. Is the person you are caring for on a bowel (poo) toileting programme? *

☐ Yes

☐ No

23. If you selected yes, please provide the start date bowel (poo) toileting programme.

24. Do you think the person's skin condition has changed in the last week? *

☐ Stayed the same as last week

☐ Better than last week

☐ Worse than last week

25. **The Ghent Global IAD Categorisation tool (GLOBIAD) sets out the categories for IAD. This involves looking at the affected skin areas of the person you are caring for and deciding which of the four categories are applicable for that person. Please can you look at the four categories for IAD and the questions set out below. Note: the IAD categories do not necessarily relate to the natural history of IAD and are not intended to suggest how IAD may develop or progress. However the tool may be helpful to record IAD.**

Does the person have any one of the four GLOBIAD categories of IAD?

(Please see below for the categories with the images) *

◯ Yes

◯ No

26. **If you answered yes that the person has IAD, please select which one of the four GLOBIAD categories that is applicable to that person.**

◯ Category 1A: Persistent redness without clinical signs of infection

◯ Category 1B: Persistent redness with clinical signs of infection

◯ Category 2A: Skin loss without clinical signs of infection

◯ Category 2B: Skin loss with clinical signs of infection

Category 1A: Persistent redness without clinical signs of infection

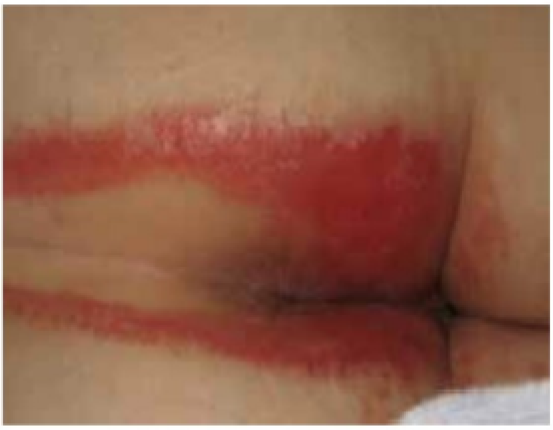

Critical criteria: Persistent redness: a variety of tones of redness may be present. Patients with darker skin tones, the skin may be paler or darker than normal, or purple in colour.

Additional criteria • Marked areas or discolouration from a previous (healed) skin defect • Shiny appearance of the skin • Macerated skin • Intact vesicles and/or bullae • Skin may feel tense or swollen at palpation • Burning, tingling, itching or pain

Category 1B: Persistent redness with clinical signs of infection

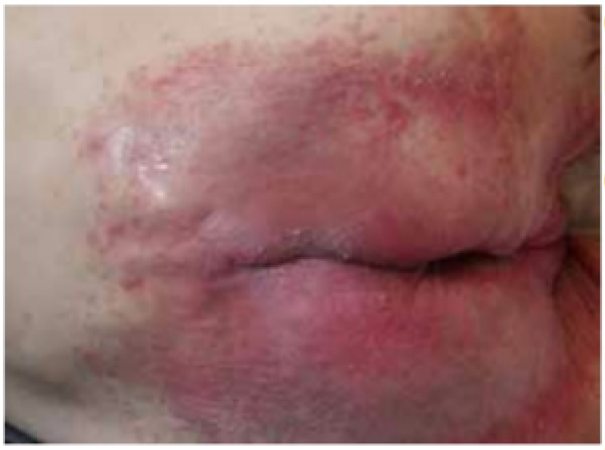

Critical criteria Persistent redness: a variety of tones of redness may be present. Patients with darker skintones, the skin may be paler or darker than normal, or purple in colour. Signs of infection: such as whitescaling of the skin (suggesting a fungal infection) or satellite lesions (pustules surrounding the lesion,suggesting a Candida albicans fungal infection).

Additional criteria • Marked areas or discolouration from a previous (healed) skin defect • Shiny appearanceof the skin • Macerated skin • Intact vesicles and/or bullae • Skin may feel tense or swollen at palpation •Burning, tingling, itching or pain

Category 2A: Skin loss without clinical signs of infection

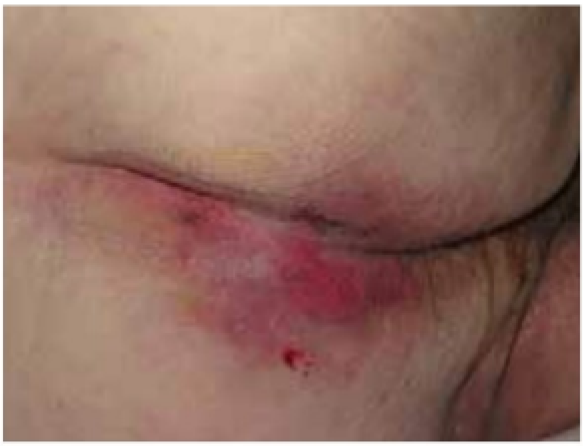

Critical criteria Skin loss: skin loss may present as skin erosion (may result from damaged/eroded vesicles or bullae), denudation or excoriation. The skin damage pattern may be diffuse.

Additional criteria • Persistent redness: a variety of tones of redness may be present. Patients with darker skin tones, the skin may be paler or darker than normal, or purple in colour • Marked areas or discolouration from a previous (healed) skin defect • Shiny appearance of the skin • Macerated skin • Intact vesicles and/or bullae • Skin may feel tense or swollen at palpation • Burning, tingling, itching or pain

Category 2B: Skin loss with clinical signs of infection

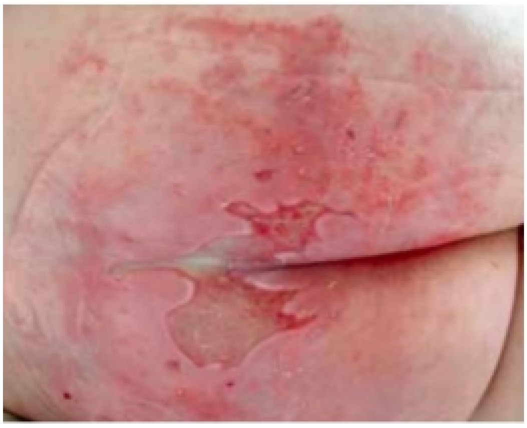

Critical criteria Skin loss: skin loss may present as skin erosion (may result from damaged/eroded vesicles or bullae), denudation or excoriation. The skin damage pattern may be diffuse.

Signs of infection: such as white scaling of the skin (suggesting a fungal infection) or satellite lesions (pustules surrounding the lesion, suggesting a Candida albicans fungal infection), slough visible in the wound bed (yellow/brown/greyish), green appearance within the wound bed (suggesting a bacterial infection with Pseudomonas aeruginosa), excessive exudate levels, purulent exudate (pus) or a shiny appearance of the wound bed.

Additional criteria • Persistent redness: a variety of tones of redness may be present. Patients with darker skin tones, the skin may be paler or darker than normal, or purple in colour • Marked areas or discolouration from a previous (healed) skin defect • Shiny appearance of the skin • Macerated skin • Intact vesicles and/or bullae • Skin may feel tense or swollen at palpation • Burning, tingling, itching or pain

Additional question

27. Is there anything else you would like to tell us about the person's skin care for incontinence/IAD?
