# Supplementary material for: PREVENTion and treatment of incontinence-associated dermatitis through a codesigned manual (PREVENT-IAD): a study protocol for a feasibility cluster randomised controlled trial with a nested process evaluation
Source: BMJ Open. 2024 Dec 23;14(12):e092338. doi: 10.1136/bmjopen-2024-092338 (PMC11667359; doi:10.1136/bmjopen-2024-092338)
Supplement: online supplemental file 5 [file bmjopen-14-12-s005.docx]

**Consent form for care home residents / community dwelling adults**
**PREVENT-IAD – phase 3**

**IRAS project ID:296167**

*Ethical Clearance Reference Number:* 23/LO/0363

**Participants Identification Number:**

**Title of Study:**

PREVENTion and Treatment of Incontinence Associated Dermatitis (IAD) through optimising care (PREVENT-IAD)
Phase 3 - Feasibility study of the IAD Manual

Thank you for considering taking part in this research. The person organising the research must explain the project to you before you agree to take part. If you have any questions arising from the Information Sheet or explanation already given to you, please ask the researcher before you decide whether to join in. You will be given a copy of this Consent Form to keep and refer to at any time.

**I confirm that I understand that by ticking/initialling each box I am consenting to this element of the study. I understand that it will be assumed that unticked/initialled boxes mean that I DO NOT consent to that part of the study. I understand that by not giving consent for any one element I may be deemed ineligible for the study.**

**Please tick or initial**

1. **I confirm that I have read and understood the information sheet dated 17/05/23 Version 2.0 for the feasibility study of the IAD Manual (phase 3 of the PREVENT-IAD study)**
2. **I have considered the information and asked questions which have been answered to my satisfaction.**
3. **I consent voluntarily to take part in this trial and that I can withdraw at any time, without having to give a reason, and that withdrawing from the trial will not affect my medical and/or social care, or other legal rights.**
4. **I agree to voluntarily participate in the following elements of the study:**

**(i) skin examinations**

**(ii) completion of the survey to assess satisfaction with the treatment**

**(iii) Observation of skin care procedures by my care-givers**

**(iv) to be interviewed about my experience of receiving care from staff using the IAD care package to prevent/treat IAD with or without my relative (if I am in this group that received the care package). The interview will take place either in person and recorded using an encrypted audio recorder or will take place online and will be audio recorded using Microsoft Teams.**

1. **I under that I can refuse to answer questions in the interview and that I can withdraw from the interview at any time, without having to give a reason, and that withdrawing from the interview will not affect my legal rights.**
2. **I agree to maintain the confidentiality of the interview discussion.**
3. **I consent to the voice recording of the interview being sent to the professional transcriber using a secured transfer system.**
4. **I understand that relevant sections of my medical notes and data collected during the study may be looked at by individuals from King’s College London and the University of Southampton research team or regulatory authorities, where it is relevant to my taking part in this research. I give permission for these individuals to have access to my records.**
5. **I understand that confidentiality and anonymity will be maintained, and it will not be possible to identify me in any research outputs.**
6. **I consent to the processing of my personal information for the purposes explained to me in the Information Sheet. I understand that such information will be handled in accordance with the terms of the UK General Data Protection Regulation and the Data Protection Act 2018.**
7. **I agree for my contact details to be stored on one of King’s College London secured storage facilities so that the researchers can invite me to participate in follow up studies to this project or in future studies of a similar nature.**
8. **I understand that the information I have submitted will be published as papers for academic journals and I wish to receive a copy of these.**
9. **I give permission for information, including the use of quotations, collected during the interview, to be used in any presentation of the findings with the understanding that I will not be identified in any of the published research study findings.**

**__________________ __________________ _________________**

**Name of Participant Date Signature**

**__________________ __________________ _________________**

**Name of Researcher Date Signature**
